# Supplementary material for: Complexity analysis of a closed-loop supply chain for power battery recycling under government subsidies
Source: PLoS One. 2025 Jun 13;20(6):e0322888. doi: 10.1371/journal.pone.0322888 (PMC12165455; doi:10.1371/journal.pone.0322888)
Supplement: Attachment — Submitted filename: Response to Reviewers.docx [file pone.0322888.s002.docx]

**Response to Reviewers**

**Complexity Analysis of a Closed-loop** **Supply Chain for Power Battery Recycling under Government Subsidies**

**Abudureheman Kadeer**^¶^ **, Xiaobin Wang**^¶^**∗, Huanying He**^¶^

We appreciate the constructive feedback provided by the reviewers. Below, we address each of the points raised and outline the improvements we plan to make to the manuscript.

**Reviewer #1:**

**1.Abstract Quantification and Scope Clarification:**

Response: We have revised the abstract to include a mention of the sensitivity analysis, emphasizing that although specific quantitative conclusions are not presented, targeted viewpoints based on the analysis have been put forth, and the scope of the research has been clearly delineated.

**2.Enhanced Motivation and Justification:**

Response: We have strengthened the introduction section by emphasizing the motivation and justification for the research. We have clearly articulated the research problem, its significance, and how our work contributes to the field.

**3.Updated Literature Review:**

Response: We have incorporated newer articles from 2024 into the literature review section to ensure comprehensiveness and up-to-date coverage of the topic.

**4.Section Numbering:**

Response: We have added numbering to all main and sub-sections to improve the organization and readability of the manuscript.

**5.Research Gaps and Innovations:**

Response: We have explicitly mentioned the research gaps and innovations that our work addresses following the literature review section.

**6.Model Advantages and Superiority:**

Response: We have clearly explained the advantages and superiority of the proposed model, highlighting its unique features and benefits over existing approaches.

**7.Detailed Sensitivity Analysis Results:**

Response: We have provided a more detailed explanation of the results from the sensitivity analyses, including an analysis of the sensitivity of profits among members, discussing their implications and how they inform our understanding of the model's behavior in a more practical context.

**8.Theoretical and Practical Significance in Conclusion:**

Response: We have revised the conclusion to reflect both the theoretical and practical significance of the manuscript, emphasizing the contributions and implications of our findings.

**9.Detailed Conclusion:**

Response: We have enhanced the conclusion section by providing more details on our contributions, limitations, the scientific value added by our paper, the applicability of our findings, and suggestions for future research.

**Reviewer #2:**

**A. Strengthening the Introduction:**

Response: We have enhanced the introduction by providing a more comprehensive background on existing issues in resiliency and sustainability. We have clearly justified the need for our method.

**B. Enhanced Literature Section:**

Response: We have incorporated the suggested references, along with other recent literature, into the literature section to better highlight research gaps, objectives, and applications of our research.

**C. Focus on Static Stackelberg and Supply Chain Efficiency:**

Response: In response to your concern, we have already conducted numerical simulations to validate three key parameters: government subsidy standards, fund return rates, and market size, primarily utilizing mathematical monotonicity (where the partial derivative is greater than 0). Additionally, we have performed sensitivity analyses on members' profits, leading to a key conclusion: In the short term, policy subsidies will increase supply chain members' profits and lower prices, but in the long term, excessive government subsidies can cause the system to enter a state of chaos, harming the profits of all parties and the overall system. Regarding your point about limited attention to specific key parameters, we acknowledge the validity of your observation. In fact, I am currently working on another article where I plan to enhance the focus on these key parameters.

**D. Variable Definition Clarity:**

Response: We will revise the variable definitions to ensure that different concepts are not represented by the same letter with only set changes. We will use distinct notation to avoid any confusion.

**E. Problem Solving Approach and Solver Details:**

Response: After extensively reviewing the literature and consulting specialized textbooks on mixed-integer programming (MIP), I acknowledge that MIP models have demonstrated effective applications in solving location and pricing problems in supply chain management. However, in the context of our current research, I believe that adopting an MIP model is not the optimal choice. Below are the specific reasons why I do not advocate for the use of an MIP model:

(1) Mismatch in Problem Nature

MIP models primarily address optimization problems, aiming to maximize or minimize a linear function subject to a series of linear constraints, with the assumption that parameters such as demand and cost are deterministic. Nevertheless, in our study, we are more concerned with the strategic interactions and decision-making sequences among multiple participants, where the key parameters are uncertain and the relationships between variables exhibit significant nonlinearity. The Stackelberg game model is particularly adept at capturing such strategic interactions and decision-making sequences, making it more suitable for analyzing our current problem.

(2) Characteristics of Decision Variables

While MIP models can accommodate both continuous and discrete (integer) decision variables, the focus of our current problem lies in the strategic choices made by participants, which often involve complex decision-making logic and dynamic changes, rather than simple quantities or counts. By utilizing nonlinear system theory, the Stackelberg game model can better handle these complex decision variables and account for the mutual influences among participants.

(3) Limitations of Solution Methods

Common solution methods for MIP models, including branch-and-bound, cutting-plane, and interior-point methods, excel at searching the feasible solution space and finding optimal or near-optimal solutions. However, in our current problem, we are interested in analyzing the strategic interactions and decision-making processes among participants, rather than merely finding an optimal solution. Furthermore, we need to analyze the stability of fixed points and the evolution of equilibrium strategies. The solution methods for the Stackelberg game model, such as backward induction, provide deeper insights into the decision-making logic and strategic choices among participants, making them more suitable for solving our current problem.

(4) Differences in Application Domains

MIP models are more focused on optimization and resource allocation problems, such as production planning, logistics planning, and resource allocation. In contrast, our current problem involves strategy formulation and decision analysis in areas such as market competition, traffic flow control, and network security. In these domains, the Stackelberg game model can better capture the strategic interactions and decision-making sequences among participants, providing more powerful strategic advice to decision-makers.

In summary, I believe that adopting an MIP model is not the best choice for our current project or problem. Instead, the Stackelberg game model can better meet our needs by capturing the strategic interactions, decision-making sequences, and complex behaviors of system evolution among participants, providing more accurate strategic advice to decision-makers. Therefore, I recommend using the Stackelberg game model for analysis and solution.

We thank the reviewers for their valuable feedback, which will significantly improve the quality and clarity of our manuscript. We will make the necessary revisions and resubmit the paper for further consideration.
